# Antitumor Effect of Saikosaponin A on Human Neuroblastoma Cells

**DOI:** 10.1155/2021/5845554

**Published:** 2021-09-01

**Authors:** Tan Cheng, Muying Ying

**Affiliations:** ^1^Joint Program of Nanchang University and Queen Mary University of London, Nanchang 330006, China; ^2^Department of Molecular Biology and Biochemistry, Basic Medical College of Nanchang University, Nanchang 330006, China

## Abstract

**Objective:**

Neuroblastoma (NB) is a highly metastatic tumor in children that develops in the sympathetic nervous system and has a low curative rate. Saikosaponin A (SSA), an active ingredient isolated from the root of Radix Bupleuri, is a natural compound with various pharmacological activities and shows good application prospects in antitumors. This study investigated the antihuman NB activity of SSA and underlying mechanisms associated with its actions.

**Materials and Methods:**

The MTT method was used to detect the activity of SSA in inhibiting human NB cell SK-N-AS proliferation. Cell morphology was observed. The flow cytometry technology was used in analyzing the cell apoptosis rate. The Transwell assay evaluated cell migration and invasion following SSA treatment, apoptosis-related protein expression, and angiogenesis-related protein expression, and EMT-related proteins were detected by western blot analysis.

**Results:**

SSA showed an inhibitory effect on SK-N-AS cells with the IC_50_ values of 14.14 *μ*M at 24 h and 12.41 *μ*M at 48 h. Results indicated that SSA has proapoptotic activity, and its proapoptotic activity is positively correlated with the Bax/Bcl-2/caspase-9/caspase-7/PARP pathway. Furthermore, SSA inhibited the invasion and migration of SK-N-AS cells via regulating the angiogenesis-related VEGFR2/Src/Akt pathway and the epithelial-mesenchymal transition- (EMT-) related protein expression.

**Conclusion:**

SSA exerts an antihuman NB effect and thus provides foundations for NB treatment.

## 1. Introduction

Neuroblastoma (NB) as a malignant tumor is a common pediatric extracranial solid tumor that develops from the sympathoadrenal lineage derived from the neural crest [1–3]. NB originates from the adrenal medulla or paravertebral sympathetic ganglia of the pelvis, abdomen, chest, or neck and has noticeably different clinical outcomes [4]. The median diagnosis age for NB is 18 months and the patients being diagnosed before their first birthday is 40%. More than 50% of diagnosed NBs are classified as “high risk” and have a low cure rate with a limited overall survival (OS) of less than 40% [5, 6]. Advancements in surgery, chemotherapy, radiation, and, most recently, immunotherapy have dramatically improved survival rates. However, a substantial number of survivors continue to experience many long-term side effects and develop various chronic health-related complications, and tumors frequently show resistance to any therapy after relapsing [6, 7]. Outcomes vary widely among patients with NB, as young children receive inadequate treatment. In contrast, older patients with metastatic disease have an approximately 50% event-free survival despite aggressive multimodal therapies with long-term severe side effects [8, 9]. Therefore, it is of great clinical and scientific significance that novel therapeutic agents associated with NB be identified. MYCN-amplified NB and non-MYCN-amplified NB are two subtypes of NB cells. MYCN is a protooncogene of the MYC family [10]. The previous studies showed that the overexpression of the MYCN gene is a leading cause of NB [11]. The MYCN-amplified NB often has a poor prognosis and occurs in the early stage. However, the mechanism of the MYCN-amplified NB is better studied than the non-MYCN-amplified NB, and the MYCN-targeting treatment could be used for the MYCN-amplified NB. The non-MYCN-amplified NB is more common, complicated, and less studied. More than 75% of NB and over 60% of poor prognosis NB are non-MYCN-amplified NB [12]. The mechanism of the non-MYCN-amplified NB remains unclear. Therefore, it is crucial to study the treatment and mechanism of non-MYCN-amplified NB.

Radix Bupleuri (RB), also named Chai Hu or Chinese Thorowax Root, is one of the Traditional Chinese Medicines. For over 2000 years, RB has been used to treat influenza, hyperlipidemia, menstrual disorders, hepatitis, and depressive disorder in Asian countries including China [13]. According to the *Pharmacopoeia of the People's Republic of China*, RB is the dry root of *Bupleurum chinense* DC. and *Bupleurum scorzonerifolium* Willd. of the Umbelliferae family. *B chinense* and *B scorzonerifolium* are widely distributed in the northern regions of China and other countries, such as Russia, Mongolia, North Korea, and Japan [14]. The main active components of RB are triterpenoid saponins, which exhibit the effects of analgesic, antiepileptic, anti-inflammatory, antifibrotic, neuromodulatory, antiviral, and antitumor activities [15]. The major active constituents of triterpenoid saponins are saikosaponins A, C, and D. Saikosaponin A (SSA) is a triterpenoid glycoside, and its structure is the ramification of pentacyclic triterpene oleane derivatives (molecular formula: C_42_H_68_O_13_, molecular weight: 781 g/mol). It has been reported that SSA has antitumor activities [16–18]. However, the effects of SSA on NB remain unclear. This study elucidated the inhibitory effects of SSA on human NB cells SK-N-AS by detecting the changes in related protein levels. Here, we revealed that SSA has an inhibitory mechanism of action on human NB cells SK-N-AS. Hence, we confirm that SSA induces apoptosis and inhibits invasion and migration in human NB cells SK-N-AS.

## 2. Materials and Methods

### 2.1. Cell Culturing

Human NB cells SK-N-AS were purchased from JFBIO (Heilongjiang, China). Human NB cells SK-N-BE were purchased from the Center for Excellence in Molecular Cell Science (CAS, Shanghai, China). Human brain glial cells (normal cell) (HBE) were purchased from Shanghai Seager Biotechnology Co., Ltd. Cells maintained in DMEM medium (Thermo Fisher) were added with 10% fetal bovine serum (FBS; Cellmax, Beijing, China), 100 U/mL penicillin, and 100 mg/mL streptomycin from Thermo Fisher. The SK-N-AS cells were cultured in the 5% CO_2_ incubator at 37°C under saturated humidity environment.

### 2.2. Cell Viability Assay

Cell viability was measured by the MTT assay (Sigma, Darmstadt, Germany). We followed the methods of Li et al. [19]. Human NB cells SK-N-AS and SK-N-BE and human normal cells HBE were cultured at 37°C and 5% CO_2_ in 96-well plates of 1 × 10^5^ cells/well. After being cultured for 24 h, the medium was replaced by a fresh medium that includes different SSA solution concentrations (0, 2.5, 5, 7.5, 10, 12.5, 15, 17.5, and 20 *μ*M SSA were used for SK-N-AS cells; 0, 2.5, 5, 7.5, 10, 15, 20, 30, and 40 *μ*M SSA were used for SK-N-BE cells; 0, 5, 10, 20, 40, 80, 120, 160, and 320 *μ*M SSA were used for HBE cells) and continuously cultured for 24 h or 48 h separately. After that, 10 *μ*L of MTT (5 mg/mL) was added to each well followed by further incubation for 4 h at 37°C. Finally, 100 *μ*L DMSO was added for dissolving the remaining formazan crystals, and the absorbance was measured at 490 nm by a Microplate Reader (RT-6000; Rayto, Shenzhen, China). The cell viability was calculated as the SSA-treated group divided by the control group and multiplied 100%.

### 2.3. Effect of SSA on Apoptosis of Human Neuroblastoma Cells SK-N-AS

Human NB cells SK-N-AS, which grew at logarithmic stages, were digested and spread on 6-well plates at the density of 1 × 10^6^/mL. Each well was cultured overnight at 37°C and 5% CO_2_. When the cell density reached 80%-90%, the medium was abandoned, and the serum-free DMEM medium was added. The concentration of SSA (Yuanye Bio-Technology, Shanghai, China) was 0, 12.5, 15, and 17.5 *μ*M, with 3 wells for each concentration. The NB cells were cultured at 37°C with 5% CO_2_. Cell morphology was observed by inverted microscopy (Leica Microsystems GmbH, Wetzlar, Germany) at 0 h, 24 h, and 48 h. Cell nuclear staining morphology was observed at 24 h according to the instructions of the Beyotime Hoechst dyeing kit [20]. Flow cytometry (Merck, Darmstadt, Germany) and Annexin V-FITC/propidium iodide (PI) double staining were performed at 24 h according to the instructions of the Beyotime Annexin V-FITC Cell Apoptosis Assay Kit (Beyotime, Shanghai, China) [21].

### 2.4. Wound Healing Assay

The methods were performed as previously reported with modifications [19, 22]. Human NB cells SK-N-AS were seeded into 6-well culture plates until 85% confluence with a complete medium. The tip of a 10 *μ*L pipette (Thermo Fisher) was used to make a horizontal and a vertical scratch on the cell monolayer of each well. The unattached cells of these wells were removed by washing with PBS 3 times. Sequently, the cells were cultured in medium containing SSA (0, 7.5, 10, or 12.5 *μ*M) for 12 h. The cells migrating toward the wound areas were observed by inverted microscopy (Leica Microsystems GmbH, Wetzlar, Germany) in various moments. The migration rate was measured by cells' migrated distance in the absence or presence of SSA.

### 2.5. Transwell Migration and Invasion Assay

The methods were performed as previously published [18, 19]. A Transwell chamber containing an 8 *μ*m pore membrane (Corning, New York, USA) was employed for migration and invasion assay. Human NB cells SK-N-AS were cultured with DMEM supplemented with 10% FBS. After reaching 85% confluence, the cells were cultured in an FBS-free medium containing SSA (0, 7.5, 10, or 12.5 *μ*M) for 24 h. The FBS-free medium does not have enough nutrition to support cell growth, avoiding increasing the size and number of cells. The cultured cells were resuspended and then diluted with serum-free medium to a density of 1 × 10^6^/mL. Before cell seeding, the upper chamber for the *invasion assay* was added with a Matrigel basement membrane matrix (BD Biosciences, Bedford, USA). Transwell chambers for the *migration assay* were not coated with Matrigel matrix. 100 *μ*L of the cell suspension was seeded into the upper chambers, and DMEM containing 10% FBS for the *migration assay* or DMEM with 20% FBS for *invasion assay* was added into the lower chamber. The cells tend to migrate and invade to the high nutrient-containing FBS serum for cell growth. Cell growth occurs after incubation at 37̊C for 24 h, and the invaded cells clinging to the lower surface of the Transwell membrane were fixed with 100% methanol and then stained with 0.1% crystal violet. The cells were counted by a microscope at five randomly selected photographing fields. The data were expressed as the average number of invaded cells in the SSA group relative to the control group.

### 2.6. Western Blot Analysis

The methods were performed as previously published [19]. Human NB cells SK-N-AS were cultured in 6-well plates at the density of 2 × 10^6^ cells/well. The NB cells were treated with SSA (0, 7.5, 10, 12.5, 15, or 17.5 *μ*M) for 24 h. After this, the NB cells were added with the RIPA buffer for cell lysing. 8,000 × g centrifugation for 15 min at 4̊C was used to collect the total protein. The total protein was determined by bicinchoninic acid assay (Beyotime, Shanghai, China). Then, 50 *μ*g of the protein was separated by 10% SDS-PAGE. The protein was then transferred to the PVDF membranes. Western blotting was performed as previously published [23]. All of the primary antibodies used were rabbit monoclonal antibodies (mAb). The mAbs of cleaved PARP, PARP, cleaved caspases (7, 9), caspases (7, 9), Bcl-2, Bax, E-cadherin, N-cadherin, Vimentin, Slug, Snail, ZO-1, p-Akt, Akt, p-Src, Src, p-VEGFR2, VEGFR2, and *β*-actin were all purchased from Cell Signaling Technology, Inc. (Danvers, MA, USA). The bands of the proteins were visualized by ECL (Applygen Technologies Inc., Beijing, China) with a Tanon 5200 Chemiluminescent Imaging System (Tanon, Shanghai, China).

### 2.7. Statistical Analysis

The software used to perform statistical analysis was SPSS (version 19.0; SPSS, Inc.) with Student's *t*-test and one-way analysis of variance (ANOVA). *p* < 0.05 was considered statistically significant. In all cases, data were expressed as mean ± standard deviation.

## 3. Results

### 3.1. Effect of SSA on Cell Viability of Human Neuroblastoma Cells SK-N-AS

**Figure 1:**
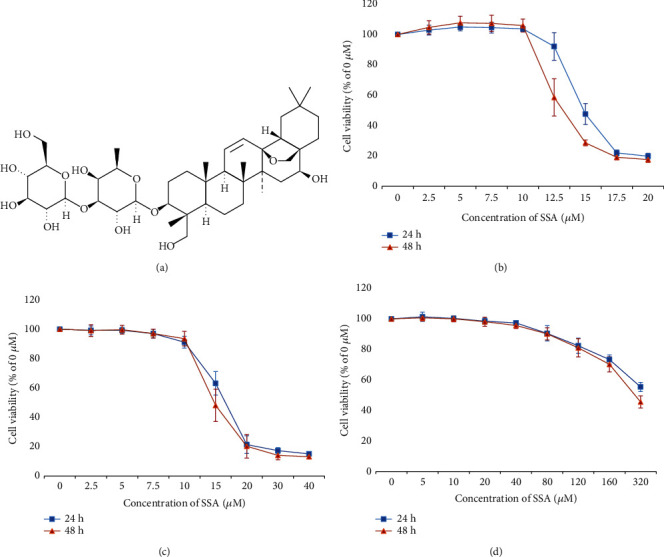
The effect of SSA on cell viability of human NB cells SK-N-AS. (a) The chemical structure of SSA. (b) Cell viability of human non-MYCN NB cells SK-N-AS treated by various concentrations of SSA (0, 2.5, 5, 7.5, 10, 12.5, 15, 17.5, or 20 *μ*M) for 24 or 48 hours. (c) Cell viability of human MYCN NB cells SK-N-BE treated by various concentrations of SSA (0, 2.5, 5, 7.5, 10, 15, 20, 30, or 40 *μ*M). (d) Cell viability of normal human cells HBE treated by various concentrations of SSA (0, 5, 10, 20, 40, 80, 120, 160, or 320). The IC_50_ values were measured (mean ± SD, *n* = 6).

Figure 1(a) displays the chemical structure of SSA. According to the MTT assay, the cytotoxicities of SSA on various cells are shown. The inhibitory effect of SSA on human NB cells indicated a particular concentration and time dependence (Figures 1(b) and 1(c)). The IC_50_ value of human NB cells SK-N-AS for 24 h was 14.14 *μ*M, and the IC_50_ value for 48 h was 12.41 *μ*M. The IC_50_ value of human NB cells SK-N-BE for 24 h was 15.48 *μ*M, and the IC_50_ value for 48 h was 14.12 *μ*M. The IC_50_ value of human normal cells HBE for 24 h was 361.3 *μ*M, and the IC_50_ value for 48 h was 283.5 *μ*M. The results showed that SSA had little cytotoxicity on normal human cells. SSA could inhibit the viability of MYCN-amplified NB cells and non-MYCN-amplified NB cells, but non-MYCN-amplified NB cells were more sensitive to SSA. In subsequent experiments, the concentration of apoptosis detection was 12.5, 15, and 17.5 *μ*M. The experimental concentrations for the detection of invasion and migration were 7.5, 10, or 12.5 *μ*M (these three concentrations had a low inhibitory effect on NB cell SK-N-AS growth, which ensured that the effects of different concentrations of SSA on SK-N-AS migration of human NB cells could be detected without affecting cell growth).

### 3.2. SSA Promotes Apoptosis of Human Neuroblastoma Cells SK-N-AS

**Figure 2:**
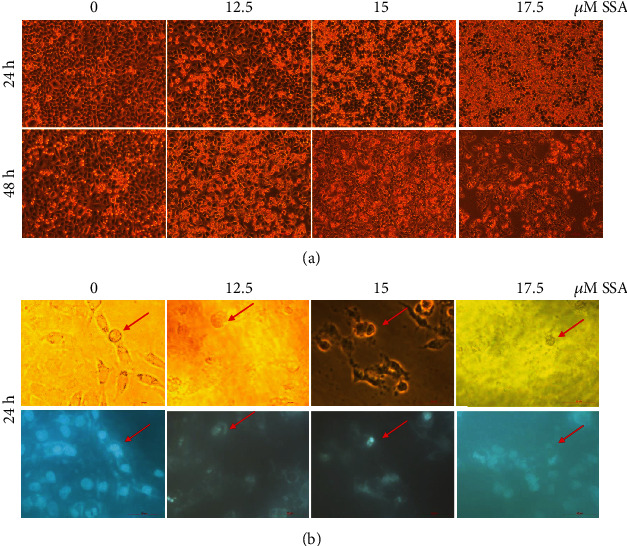
The morphological changes of human neuroblastoma cells and cell nucleus after treatment with SSA. (a) Morphological changes of human NB cells SK-N-AS treated with different concentrations of SSA (0, 12.5, 15, and 17.5 *μ*M) for 24 h or 48 h under a light microscope (100x). (b) Cell nuclear morphological changes of human NB cells SK-N-AS treated with SSA by Hoechst 33258 staining in the same field observed under a light and fluorescent microscope (400x). Nuclei indicated by the arrows were round, irregular, condensed, and fragmented by treatment with SSA at 0 *μ*M, 12.5 *μ*M, 15 *μ*M, and 17.5 *μ*M, respectively.

**Figure 3:**
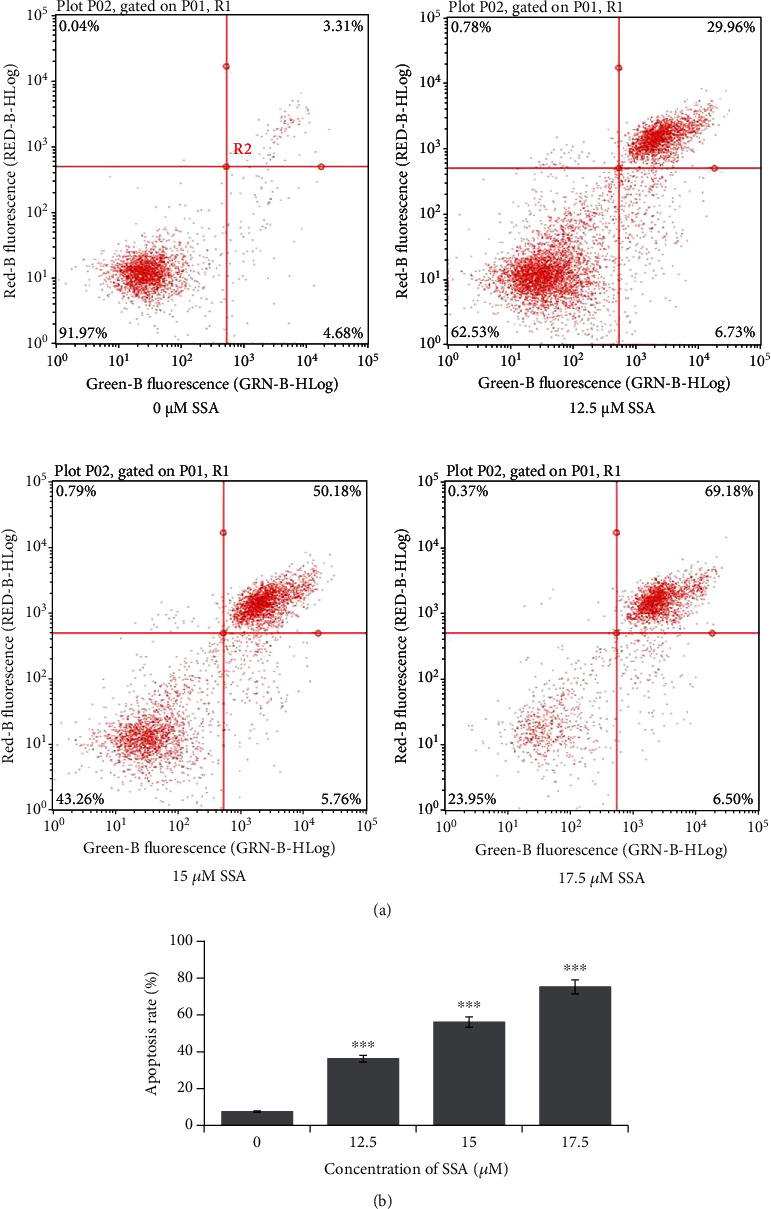
Apoptosis condition of human NB cells SK-N-AS with the treatment of different concentrations of SSA (0, 12.5, 15, and 17.5 *μ*M) measured by (a) flow cytometry and the corresponding (b) bar graph, which represents the apoptosis rate of SK-N-AS (mean ± SD, *n* = 3). ^∗^*p* < 0.05, ^∗∗^*p* < 0.01, and ^∗∗∗^*p* < 0.001 vs. control.

**Figure 4:**
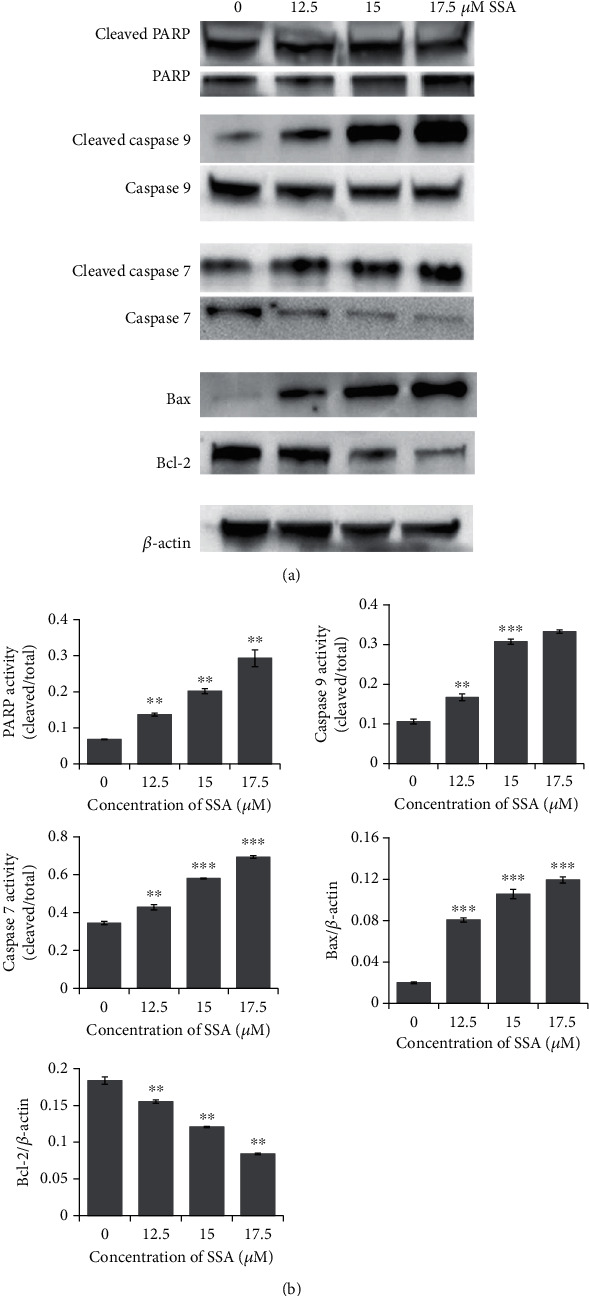
Apoptosis-related protein expression after treatment with different concentrations of SSA (0, 12.5, 15, and 17.5 *μ*M): (a) western blot and (b) quantifications (mean ± SD, *n* = 3). ^∗^*p* < 0.05, ^∗∗^*p* < 0.01, and ^∗∗∗^*p* < 0.001 vs. control.

Human NB cells SK-N-AS were incubated with SSA (0, 12.5, 15, or 17.5 *μ*M) after 24 h or 48 h. The effect of SSA on the apoptosis of these cells was evaluated by observation of the morphological changes of NB cells and cell nuclear morphological changes with Hoechst 33258 staining and flow cytometry with Annexin V-FITC/PI staining. The expression of apoptosis-related proteins after SSA treatment was analyzed by western blotting. After SSA treatment, human NB cells showed specific morphological changes of apoptosis, including cell volume reduction and abnormal forms of nuclei (Figure 2(a)). The nuclei of the cells were round without the influence of SSA. When the concentration of SSA increased, the shape of nuclei became irregular. The chromatin became condensed or fragmented (Figure 2(b)). The results of Annexin V-FITC/PI double staining indicated that the apoptotic rate of human NB cells significantly increased after SSA treatment. The apoptotic rate was positively correlated with the drug concentration (Figures 3(a) and 3(b)). To further elucidate this, expressions of apoptosis-related proteins after SSA treatment were analyzed by western blotting. PARP activity, caspase-9 activity, caspase-7 activity, and Bax expression level were increased; the Bcl-2 expression level was decreased; and the expression changes were correlated with drug administration concentration (Figures 4(a) and 4(b)). The results indicated that SSA was able to inhibit NB cells growth by activating the apoptosis pathway. The expression of substrate PARP, cleaved caspase-9, and cleaved caspase-7 in the exogenous pathway. The expression of proapoptotic proteins Bax and antiapoptotic proteins Bcl-2 was influenced by SSA in the endogenous pathway.

### 3.3. SSA Inhibits Migration and Invasion of Human Neuroblastoma Cells SK-N-AS

**Figure 5:**
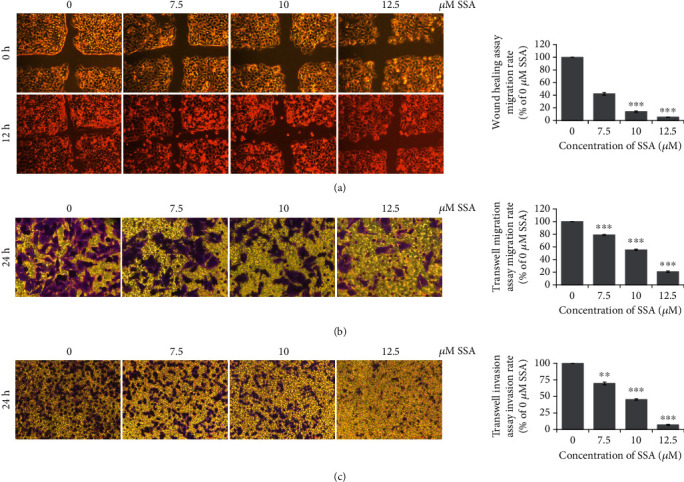
Effects of SSA on migration and invasion ability of human neuroblastoma cells SK-N-AS. Photographs and quantifications of (a) wound healing assay treated by different concentrations and times of SSA (0, 7.5, 10, and 12.5 *μ*M) (100x). Photographs and quantifications of (b) Transwell migration assay (200x) and (c) Transwell invasion treated by different concentrations of SSA (100x) (mean ± SD, *n* = 3). ^∗^*p* < 0.05, ^∗∗^*p* < 0.01, and ^∗∗∗^*p* < 0.001 vs. control.

**Figure 6:**
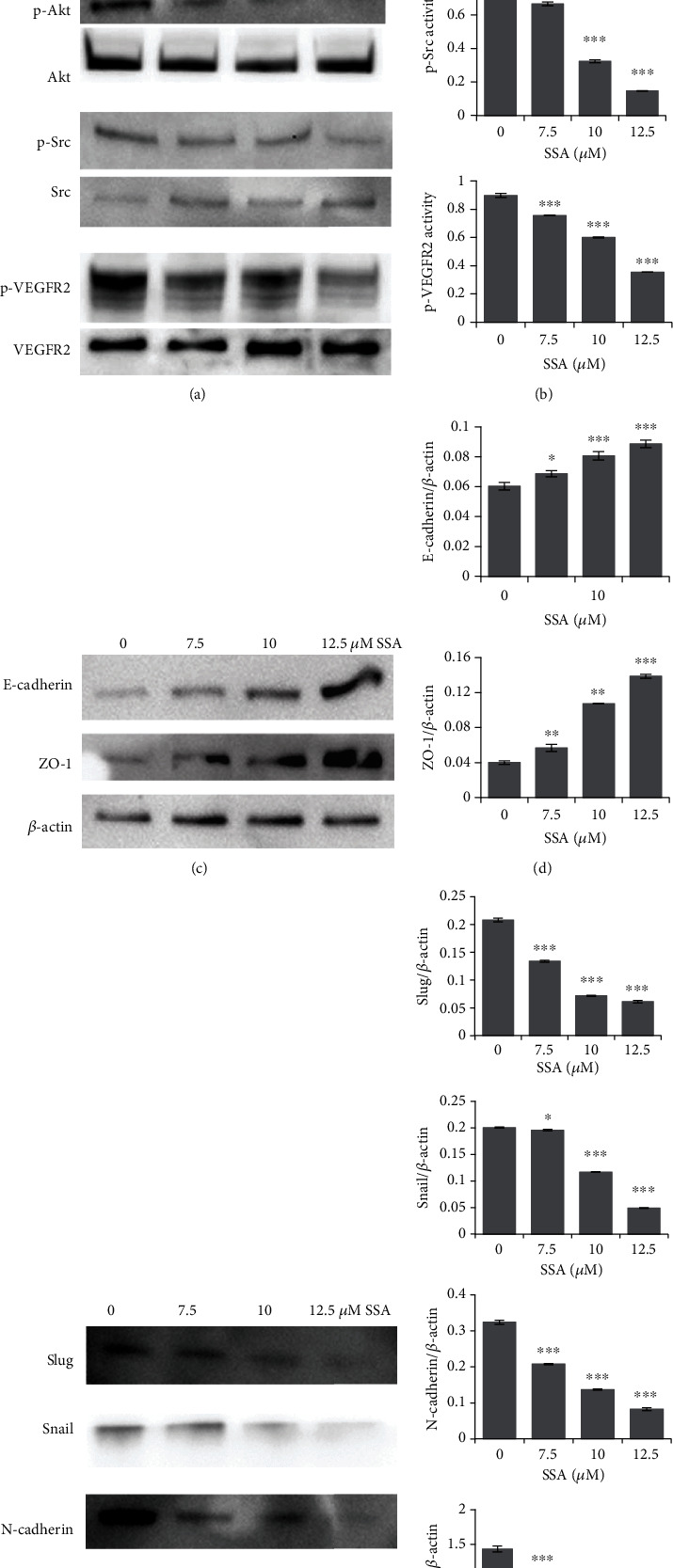
Expression of migration and invasion-related proteins after treatment with different concentrations of SSA. Expression of (a) angiogenesis-related proteins and EMT-related proteins, (c) epithelial markers, and (e) mesenchymal markers after treatment with different concentrations of SSA (0, 12.5, 15, and 17.5 *μ*M). The expressions of (b) angiogenesis proteins, (d) epithelial markers, and (f) mesenchymal markers were quantified with bar graphs (mean ± SD, *n* = 3). ^∗^*p* < 0.05, ^∗∗^*p* < 0.01, and ^∗∗∗^*p* < 0.001 vs. control.

**Figure 7:**
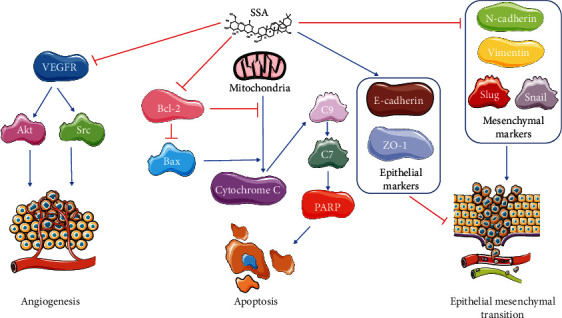
The effect of SSA on human non-MYCN NB cells. SSA could inhibit NB cells' angiogenesis by inhibiting the VEGFR/Src/Akt pathway. SSA could induce NB cells apoptosis via the Bax/cytochrome C/C9/C7/PARP pathway by inhibiting Bcl-2. SSA could inhibit EMT by inhibiting the mesenchymal markers and inducing the epithelial markers.

To assess the capacity of SSA in inhibiting migration and invasion of human NB cells SK-N-AS, wound healing assay, Transwell migration, and invasion assays were carried out. The expressions of invasion and migration-related proteins are measured and quantified by western blotting. According to the MTT assay results, 0, 7.5, 10, and 12.5 *μ*M SSA were selected for this performance. The results of the wound healing and Transwell assay showed that SSA significantly suppressed the invasion and migration of human NB cells SK-N-AS dose-dependently (Figures 5(a)–5(c)). p-VEGFR2, p-Akt, and p-Src expression levels were decreased after SSA treatment, and the expression changes were related to the concentration of administration (Figures 6(a) and 6(b)). After SSA treatment, the expression levels of Vimentin, N-cadherin, Slug, and Snail were decreased, while the expression levels of ZO-1 and E-cadherin were increased, and the change of expression was related to the concentration of administration (Figures 6(c)–6(f)). These results suggest that SSA has the ability to inhibit the invasion and migration of human NB cells SK-N-AS by regulating the intracellular angiogenesis-related VEGFR2/Src/Akt pathway and the EMT-related proteins. Upon our investigation, SSA, a natural chemical compound, exhibited antitumor effects on non-MYCN NB by inhibiting cell proliferation, migration, and invasion. (Figure 7).

## 4. Discussion

As the second common extracranial malignant tumor in children, NB is the most common solid tumor with high risk and low cure rate for fatal demise [24]. SSA isolated from Radix Bupleuri exerts antitumor activities [25]. Therefore, the antitumor activity of SSA on human NB cells was investigated in this study. The results indicated that SSA could inhibit human NB cells' proliferation, migration, and invasion.

Antiapoptosis is a common feature in cancers [26–28]. The caspase protein family contains cysteine residues capable of specific cleavage of peptide bonds associated with target aspartic acid residues. Caspase-9 is an essential protease during the apoptosis process. The active form of caspase-9 can hydrolyze caspase-7 to activate it. Activated caspase-7 hydrolyzes PARP to produce cleaved PARP, which is a prerequisite for apoptosis [29]. Bcl-2 can suppress caspase activation by inhibiting the mitochondria releasing cytochrome C, thereby inhibiting apoptosis driven by cytochrome C, activating the caspase-9 pathway [30, 31]. Bax has a proapoptotic effect, and its activation could induce the release of cytochrome C from the mitochondria [32–35]. Bax protein remains functional, but most of the Bax activity is neutralized by antiapoptotic Bcl-2 protein, which is usually overexpressed. Therefore, Bax activation is effective in tumor therapy [36]. These results suggested that SSA could promote the apoptosis of SK-N-AS cells by regulating the intracellular caspase protein family via the Bax/Bcl-2/caspase-9/caspase-7/PARP pathway.

Angiogenesis and epithelial-mesenchymal transition (EMT) are two main causes of cancer migration and invasion [37]. Vascular endothelial growth factor receptor 2 (VEGFR2) signal transduction is essential in the angiogenesis progress in vivo [38]. When binding to ligands, VEGFR2 autophosphorylates and is activated. VEGFR2 activation during angiogenesis results in signal transduction through multiple downstream kinase signaling pathways, including Akt and Src [39]. Src and Akt activation promote cell survival, proliferation, growth, and changes in cell metabolic pathways [40]. Therefore, expression levels of p-VEGFR2, p-Src, and p-Akt increased when the cells generated blood vessels. These results suggest that SSA can inhibit both invasion and migration of human NB cells SK-N-AS by regulating the intracellular angiogenesis-related VEGFR2/Src/Akt pathway. Cadherin mediates calcium-dependent intercellular adhesion and plays a crucial role in normal tissue development. E-cadherin is considered to be an active inhibitor of the growth and invasion of many cancer cells [41]. Recent studies have shown that the upregulation expression of N-cadherin and the downregulation expression of E-cadherin are hallmarks of EMT [42]. Vimentin is one of the major constituents of the intermediate filament protein family and helps coordinate different signal transduction pathways, inducing EMT in tumor cells [43]. Zonula occludens-1 (ZO-1) is vital in tight junction, and loss of the ZO-1 protein induces EMT [44]. Slug and Snail can travel between the cytoplasm and nucleus to inhibit the expression of the E-cadherin gene, thereby triggering EMT [45–48]. Therefore, when EMT occurs, the expression levels of Vimentin, N-cadherin, Slug, and Snail increase, while the expression levels of ZO-1 and E-cadherin decrease. Our results suggest that SSA can inhibit the metastasis and invasion of human NB cells SK-N-AS by regulating EMT-related proteins.

The results showed that SSA might inhibit the invasion ability of SK-N-AS in human NB cells. Meanwhile, western blot results also confirmed that SSA can affect the expression of angiogenesis-related proteins and epithelial-mesenchymal cell transition-related proteins, suggesting that SSA may inhibit the invasion of SK-N-AS in human NB cells.

## 5. Conclusions

The present results showed that SSA could inhibit human NB cells' proliferation, migration, and invasion. Apoptosis in human NB cells SK-N-AS was through the Bax/Bcl-2/caspase-9/caspase-7/PARP pathway. SSA demonstrated anti-invasion and antimetastasis by regulating the VEGFR2/SRC/AKT pathway and EMT-related proteins, which involved E-cadherin, N-cadherin, Vimentin, ZO-1, Slug, and Snail. The results above present foundations for further investigations of SSA as an antitumor agent for NB treatment.

## Data Availability

The data used to support the findings of this study are available from the corresponding authors upon request.

## References

[1] Brodeur G. M. (2003). Neuroblastoma: biological insights into a clinical enigma. *Nature Reviews. Cancer*.

[2] Gatta G., Ferrari A., Stiller C. A. (2012). Embryonal cancers in Europe. *European Journal of Cancer*.

[3] Ward E., DeSantis C., Robbins A., Kohler B., Jemal A. (2014). Childhood and adolescent cancer statistics, 2014. *CA: a Cancer Journal for Clinicians*.

[4] Maris J. M. (2010). Recent advances in neuroblastoma. *The New England Journal of Medicine*.

[5] Matthay K. K., Maris J. M., Schleiermacher G. (2016). Neuroblastoma. *Nature Reviews Disease Primers*.

[6] Maris J. M., Hogarty M. D., Bagatell R., Cohn S. L. (2007). Neuroblastoma. *The Lancet*.

[7] Castleberry R. P. (1997). Neuroblastoma. *European Journal of Cancer*.

[8] Ladenstein R., Pötschger U., Pearson A. D. J. (2017). Busulfan and melphalan versus carboplatin, etoposide, and melphalan as high- dose chemotherapy for high-risk neuroblastoma (HR-NBL1/SIOPEN): an international, randomised, multi-arm, open-label, phase 3 trial. *The Lancet Oncology*.

[9] Elzembely M. M., Dahlberg A. E., Pinto N. (2019). Late effects in high-risk neuroblastoma survivors treated with high-dose chemotherapy and stem cell rescue. *Pediatric Blood & Cancer*.

[10] Masserot C., Liu Q., Nguyen E. (2016). WT1 expression is inversely correlated with MYCN amplification or expression and associated with poor survival in non-MYCN-amplified neuroblastoma. *Molecular Oncology*.

[11] Otte J., Dyberg C., Pepich A., Johnsen J. I. (2021). MYCN function in neuroblastoma development. *Frontiers in Oncology*.

[12] Lee E., Lee J. W., Lee B. (2020). Genomic profile of MYCN non-amplified neuroblastoma and potential for immunotherapeutic strategies in neuroblastoma. *BMC Medical Genomics*.

[13] Yang F., Dong X., Yin X., Wang W., You L., Ni J. (2017). Radix Bupleuri: a review of traditional uses, botany, phytochemistry, pharmacology, and toxicology. *BioMed Research International*.

[14] Li X., Liu R., Zhang L., Jiang Z. (2017). The emerging role of AMP-activated protein kinase in cholestatic liver diseases. *Pharmacological Research*.

[15] Vinet L., Zhedanov A. (2011). A ‘missing’ family of classical orthogonal polynomials. *Journal of Physics A: Mathematical and Theoretical*.

[16] Kim B. M. (2018). The role of saikosaponins in therapeutic strategies for age-related diseases. *Oxidative Medicine and Cellular Longevity*.

[17] Kang S. J., Lee Y. J., Kang S. G. (2017). Caspase-4 is essential for saikosaponin a-induced apoptosis acting upstream of caspase-2 and *γ*-H2AX in colon cancer cells. *Oncotarget*.

[18] Wang Y., Zhao L., Han X. (2020). Saikosaponin A inhibits triple-negative breast cancer growth and metastasis through downregulation of CXCR4. *Frontiers in Oncology*.

[19] Jiang Q., Pan Y., Cheng Y., Li H., Liu D., Li H. (2016). Lunasin suppresses the migration and invasion of breast cancer cells by inhibiting matrix metalloproteinase-2/-9 via the FAK/Akt/ERK and NF-*κ*B signaling pathways. *Oncology Reports*.

[20] Cardinale A. (2005). Basic Cell Culture Protocols. *Allergy & Clinical Immunology International - Journal of the World Allergy Organization*.

[21] Métézeau P. (1988). Practical flow cytometry. *Annales de l'Institut Pasteur / Immunologie*.

[22] Liang C. C., Park A. Y., Guan J. L. (2007). In vitro scratch assay: a convenient and inexpensive method for analysis of cell migration in vitro. *Nature Protocols*.

[23] Zhou R., Xu L., Ye M., Liao M., Du H., Chen H. (2014). Formononetin inhibits migration and invasion of MDA-MB-231 and 4T1 breast cancer cells by suppressing MMP-2 and MMP-9 through PI3K/AKT signaling pathways. *Hormone and Metabolic Research*.

[24] Park J. R., Eggert A., Caron H. (2008). Neuroblastoma: biology, prognosis, and treatment. *Pediatric Clinics of North America*.

[25] Zhao X., Liu J., Ge S. (2019). Saikosaponin A inhibits breast cancer by regulating Th1/Th2 balance. *Frontiers in Pharmacology*.

[26] Hanahan D., Weinberg R. A. (2011). Hallmarks of cancer: the next generation. *Cell*.

[27] Yip K. W., Reed J. C. (2008). Bcl-2 family proteins and cancer. *Oncogene*.

[28] Baker S. J., Reddy E. P. (1998). Modulation of life and death by the TNF receptor superfamily. *Oncogene*.

[29] Zhao G., Zhu Y., Eno C. O. (2014). Activation of the proapoptotic Bcl-2 protein Bax by a small molecule induces tumor cell apoptosis. *Molecular and Cellular Biology*.

[30] Chipuk J. E., Green D. R. (2008). How do BCL-2 proteins induce mitochondrial outer membrane permeabilization?. *Trends in Cell Biology*.

[31] Danial N. N., Korsmeyer S. J. (2004). Cell death: critical control points. *Cell*.

[32] Lindsten T., Ross A. J., King A. (2000). The combined functions of proapoptotic Bcl-2 family members Bak and Bax are essential for normal development of multiple tissues. *Molecular Cell*.

[33] Wei M. C., Zong W. X., Cheng E. H. Y. (2001). Proapoptotic BAX and BAK: a requisite gateway to mitochondrial dysfunction and death. *Science*.

[34] Walensky L. D., Gavathiotis E. (2011). BAX unleashed: the biochemical transformation of an inactive cytosolic monomer into a toxic mitochondrial pore. *Trends in Biochemical Sciences*.

[35] Sonoda Y., Matsumoto Y., Funakoshi M., Yamamoto D., Hanks S. K., Kasahara T. (2000). Anti-apoptotic Role of Focal Adhesion Kinase (FAK). *The Journal of Biological Chemistry*.

[36] Suzuki M., Youle R. J., Tjandra N. (2000). Structure of bax: coregulation of dimer formation and intracellular localization. *Cell*.

[37] Sánchez-Tilló E., Liu Y., de Barrios O. (2012). EMT-activating transcription factors in cancer: beyond EMT and tumor invasiveness. *Cellular and Molecular Life Sciences*.

[38] Simons M., Gordon E., Claesson-Welsh L. (2016). Mechanisms and regulation of endothelial VEGF receptor signalling. *Nature Reviews. Molecular Cell Biology*.

[39] Jin Z. G., Ueba H., Tanimoto T., Lungu A. O., Frame M. D., Berk B. C. (2003). Ligand-independent activation of vascular endothelial growth factor receptor 2 by fluid shear stress regulates activation of endothelial nitric oxide synthase. *Circulation Research*.

[40] Claesson-Welsh L., Welsh M. (2013). VEGFA and tumour angiogenesis. *Journal of Internal Medicine*.

[41] Serrano-Gomez S. J., Maziveyi M., Alahari S. K. (2016). Regulation of epithelial-mesenchymal transition through epigenetic and post-translational modifications. *Molecular Cancer*.

[42] Maeda M., Johnson K. R., Wheelock M. J. (2005). Cadherin switching: essential for behavioral but not morphological changes during an epithelium-to-mesenchyme transition. *Journal of Cell Science*.

[43] Satelli A., Li S. (2011). Vimentin in cancer and its potential as a molecular target for cancer therapy. *Cellular and Molecular Life Sciences*.

[44] Sugrue S. P., Zieske J. D. (1997). ZO1 in corneal epithelium: association to the zonula occludens and adherens junctions. *Experimental Eye Research*.

[45] Mendez M. G., Kojima S., Goldman R. D. (2010). Vimentin induces changes in cell shape, motility, and adhesion during the epithelial to mesenchymal transition. *The FASEB Journal*.

[46] Liu J., Li J., Li P. (2017). Loss of DLG5 promotes breast cancer malignancy by inhibiting the Hippo signaling pathway. *Scientific Reports*.

[47] Wang Y., Shi J., Chai K., Ying X., Zhou B. (2013). The role of snail in EMT and tumorigenesis. *Current Cancer Drug Targets*.

[48] Dhasarathy A., Phadke D., Mav D., Shah R. R., Wade P. A. (2011). The transcription factors snail and slug activate the transforming growth factor-beta signaling pathway in breast cancer. *PLoS One*.

